# Trauma Scores and Their Prognostic Value for the Outcome Following Pediatric Polytrauma

**DOI:** 10.3389/fped.2021.721585

**Published:** 2021-09-03

**Authors:** Danielle S. Wendling-Keim, Anja Hefele, Oliver Muensterer, Markus Lehner

**Affiliations:** ^1^Department of Pediatric Surgery, Dr. von Hauner Children's Hospital, Ludwig-Maximilians-University, Munich, Germany; ^2^Department of Pediatric Surgery, Children's Hospital, Luzerner Kantonsspital, Lucerne, Switzerland

**Keywords:** glasgow coma scale, pediatric trauma score, national advisory committee for aeronautics, pediatric polytrauma, injury severity score, outcome

## Abstract

**Purpose:** The management and prognostic assessment of pediatric polytrauma patients can pose substantial challenges. Trauma scores developed for adults are not universally applicable in children. An accurate prediction of the severity of trauma and correct assessment of the necessity of surgical procedures are important for optimal treatment. Several trauma scores are currently available, but the advantages and drawbacks for use in pediatric patients are unclear. This study examines the value of the trauma scores Injury Severity Score (ISS), Pediatric Trauma Score (PTS), National Advisory Committee for Aeronautics (NACA), and Glasgow Coma Score (GCS) for the assessment of the polytraumatized child.

**Methods:** In a retrospective study, 97 patients aged 0–17 years who presented with polytrauma and an ISS ≥16 in the trauma bay were included in the study. Patient records including radiological studies were analyzed. Pathological imaging findings and emergency surgery were assessed as outcome variables and the predictive value of the trauma scores were analyzed using receiver operator characteristic (ROC) curves. Statistical significance was set at an alpha level of *P* ≤ 0.05.

**Results:** In this study, 35 of the 97 studied children had pathological cranial computed findings. These either underwent craniectomy or trepanation or a parenchymal catheter was placed for intracranial pressure monitoring. Abdominal trauma was present in 45 patients, 16 of which were treated surgically. Forty-three patients arrived with thoracic injuries, 10 of which received a thoracic drainage. One child underwent an emergency thoracotomy. Predictive accuracy for emergency surgery calculated using receiver-operator characteristic (ROC) curves was highest for ISS and NACA scores (0,732 and 0.683, respectively), and lower for GCS (0.246) and PTS (0.261).

**Conclusion:** In our study cohort, initial ISS and NACA scores better predicted operative interventions and outcome than PTS or GCS for polytraumatized pediatric patients.

## Introduction

Pediatric trauma is the leading cause of death among children (https://www.cdc.gov/injury/wisqars/pdf/leading_causes_of_death_by_age_group_2018-508.pdf). Although the management of pediatric trauma patients follows the same sequence of checking for airway, breathing, circulation (e.g., ATLS) as in adults, some aspects require specialized care due to anatomic and physiologic age-dependent differences. Knowledge of these special characteristics is crucial to avoid errors during diagnostic and therapeutic steps (1–3) and to initiate prompt diagnosis and treatment. For the speedy assessment and appropriate allocation of resources with multiply injured patients, trauma scoring systems have been established. The Glasgow Coma Scale (GCS) is most commonly applied to measure the impairment of consciousness by assessing the ocular, motor, and verbal reaction of the patient (4–8). The Injury Severity Score (ISS), on the other hand, analyses six different body systems and weighs the seriousness of the trauma by including the three worst injuries (9). The Pediatric Trauma Score (PTS) not only includes skeletal injuries, cutaneous wounds, and damage to the central nervous system, but also physiological parameters, such as body weight, systolic blood pressure, and airway status (10, 11). The National Advisory Committee for Aeronautics (NACA) score is frequently used in the prehospital phase and classifies the severity of the injury (12).

Trauma scoring systems relevant for the adult trauma patient have been criticized for their relevance in assessing the multiply injured child (13). However, the value of various pediatric scores, such as the Pediatric Trauma Score (PTS) as a prognostic factor for the outcome after polytrauma in the pediatric patient, has been controversial (14–16). The perfect instrument to estimate pediatric trauma is still a matter of debate.

Therefore, it was the goal of this study to analyze and evaluate different trauma scores and parameters to correlate these to the outcome and relevance after major trauma in children treated in a university-hospital based Level 1 pediatric trauma center.

## Patients and Methods

Patients from birth up until 17 years of age who presented with polytrauma at our pediatric level one trauma center over a 12-year study period were retrospectively included from the hospital's electronic archive. Burn patients were excluded. The severity of the polytrauma was classified using different scoring systems (PTS, ISS, NACA, and GCS). The pediatric Glasgow Coma scale was used for children up to the age of 5 years (17–20). For analysis, we grouped the patients according to their age in Newborns, infants, toddlers, children aged 6–12 years and teenagers aged 13–18 years of age. Only patients with an ISS ≥ 16 were included in the study according to previous work (21, 22).

The sensitivity and specificity of the different trauma scores and parameters were evaluated. Their validity in predicting operative intervention and outcome was compared using receiver operating characteristic (ROC) curves. Statistical analysis was performed using IBM SPSS Statistics 20.0. The Pearson's χ^2^-test and the *t*-test were applied. In cases of *n* < 5, the Fisher exact test was applied. For comparison of more than two groups, Analysis of Variance (ANOVA) was applied. The area under the ROC curve (AUC) was calculated. The standard deviation was indicated with +/- following the mean. Statistical significance was set at an alpha level of *P* ≤ 0.05.

## Results

### Study Population

**Figure 1:**
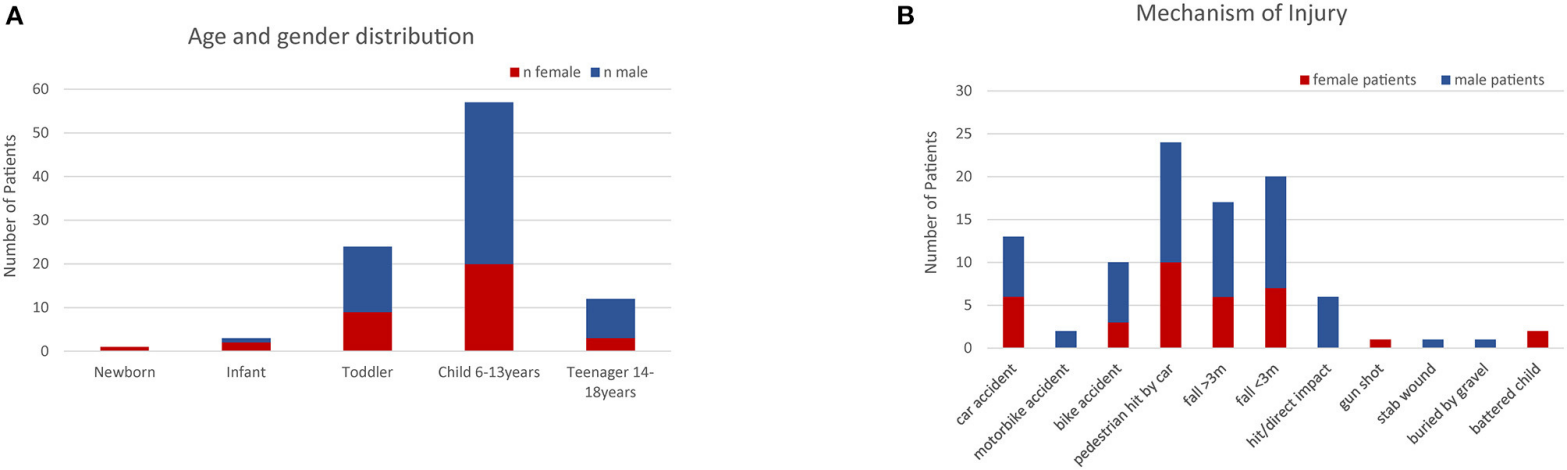
**(A)** Age and gender distribution of the patients included in the study. **(B)** Mechanisms of injury. The most frequent cause of polytrauma in this study were traffic accidents and falls.

We identified 97 patients who fulfilled the inclusion criteria. The age and gender distributions of the patients are shown in Figure 1A. The patients were stratified by age into five groups (Figure 1A). The group of children aged 6–12 years made up the largest cohort of patients, and the second largest group was toddlers and preschool children from 1 to 5 years. Male patients were more prevalent than female patients (65 and 35%, respectively). The mean age of the study population was 8.0 years with a standard deviation of 4.1 years.

Concerning the injury mechanism, 95 of the 97 patients presented with blunt trauma. One of the remaining four patients was injured by a gunshot, another by a knife. The most frequent trauma mechanisms leading to polytrauma were traffic accidents and falls (Figure 1B). Two patients were victims of battered child syndrome.

The majority of patients (64%) included in the study were transferred directly to our institution from the trauma scene, whereas 36% were transported from other hospitals after initial stabilization. We observed that the mode of transportation was significantly associated with the ISS score. The mean ISS was 31.7+/-9.1 in patients who were transported by helicopter whereas patients transferred by a ground-based doctor-accompanied ambulance had a mean ISS of 22.3 +/-10.2 (*p* = 0.0340).

### Diagnostics and Intervention/Surgery

The diagnostic imaging tools most often applied within the 1st hour after the arrival in the trauma room were Focused Assessment with Sonography for Trauma (FAST), cranial computed tomography (CT), and conventional plain-film radiographs.

### Traumatic Brain Injury

Cranial computed tomography (CT) was performed in 60 cases. Furthermore, four patients had a whole-body spiral CT, and two children had a cranial ultrasound. Out of the 65 patients who underwent cranial imaging, 35 patients were found to have pathological findings, including cerebral edema (*n* = 13), epidural hematoma (*n* = 13), and subdural hematoma (*n* = 8). An emergency craniectomy due to intracerebral hemorrhage or edema was performed in 10 patients. Four patients had a trepanation within 24 h after trauma. Intracranial pressure monitoring catheters were placed in 31 patients.

### Abdominal and Thoracic Trauma

Focused abdominal sonography for trauma (FAST, *n* = 65) and more extensive abdominal sonography (*n* = 6) identified 28 patients with free abdominal or pleural fluid. Five of these patients had an immediate laparotomy, and five more patients had surgical interventions during their hospital stay. In 28 patients, neither FAST nor extensive abdominal sonography was performed. In the majority of these cases, patients were either transferred from another hospital or other imaging methods were primarily used (cranial CT, trauma CT, thoracic X-ray). Unstable patients underwent emergency surgery without prior imaging. Most FAST sonographies were performed within the 1st hour after arrival in the emergency room, and immediately on arrival in seven patients after falls from higher than 3 m or other direct trauma (Table 1). Injuries of the chest and the lungs (contusion, pleural effusion, pneumothorax, and hemothorax) occurred in 43 cases (Table 1), prompting the team to place a chest tube in 10 cases. One case underwent emergency thoracotomy after a gunshot to the chest. The severity of the thoracic trauma according to the thoracic component of the ISS corresponded with mortality (*p* = 0.04).

**Table 1:** Mechanism leading to pathological findings in FAST (focused abdominal sonography for trauma) and thoracic injuries.

**Patients with pathological FAST findings**	
**Mechanis of injury**	***n***
Car accident	3
Bicylce accident	6
Pedestrian hit by car	6
Fall >3 m	3
Fall <3 m	6
Hit	2
Stab	1
Other	1
**Patients with thoracic injuries**	
**Type of injury**	***n***
Lung contusion	15
Pleural effusion	8
Pneumothorax	12
Hemothorax	3
Atelectasis	3
Other	5

### Fractures

Fractures were located in the facial skull cap and the remaining skull in 27 and 26 cases, respectively. Fractures of the long bones were found in 23 cases. Only four of these fractures underwent urgent surgery after admission to the emergency room. The primary indication for surgery was an open fracture. Sixteen patients with fractures were operated on in a delayed fashion within the course of their hospital stay.

### Intubation and CPR

A total of 28 patients (28.9%) were intubated at the trauma scene. Two patients underwent cardiopulmonary resuscitation (CPR). One infant presented with a GCS of 3 and died from traumatic brain injury (TBI) on the day of the injury after a car accident. A toddler who was intubated at the scene had a GCS of 3 after a fall from <3 m height. He was transferred to our institution 4 days after the injury. He was discharged for rehabilitation after 12 days.

### Outcome

The majority of patients (60.8%) were discharged home, while 25.8% of patients were transferred to a rehabilitation center and eight patients were treated in another hospital closer to their home. Five children died (5.2%), three of whom presented with traumatic brain injury. Six patients (6%) suffered from organ failure, including cardiovascular failure in five cases as well as kidney failure in three cases, and disseminated intravascular coagulation in one case. Furthermore, three patients developed sepsis, and two patients developed multi-organ failure. However, the main predictors of lethality after polytrauma were the occurrence of traumatic brain injury (*p* = 0.0001) and severe thoracic injury (*p* = 0.04). Of the patients who did not survive, five died within the first 24 h after trauma.

### Trauma Scoring Systems

Table 2 shows the scores of the patients according to the Glasgow Coma Scale (GCS) and the pediatric GCS, respectively, Injury Severity Scale (ISS), Pediatric Trauma Score (PTS), and National Advisory Committee for Aeronautics score (NACA score).

**Table 2:** Different trauma scores GCS, ISS, and PTS of the patients in this study are listed here.

**Trauma score**	**Number of patients**
	**Initial assessment at trauma site**
GCS 13–15	54
GCS 9–12	15
GCS 3–8	19
GCS n.a.	9
ISS 16–20	27
ISS 21–30	45
ISS 31–40	9
ISS 41–50	13
ISS 51–60	3
PTS−5	1
PTS 0	2
PTS 1	3
PTS 2	2
PTS 3	7
PTS 4	5
PTS 5	1
PTS 6	5
PTS 7	13
PTS 8	8
PTS 9	11
PTS 10	7
PTS 11	10
PTS 12	19
NACA 3	11
NACA 4	22
NACA 5	29

### Initial Scoring With NACA

**Figure 2:**
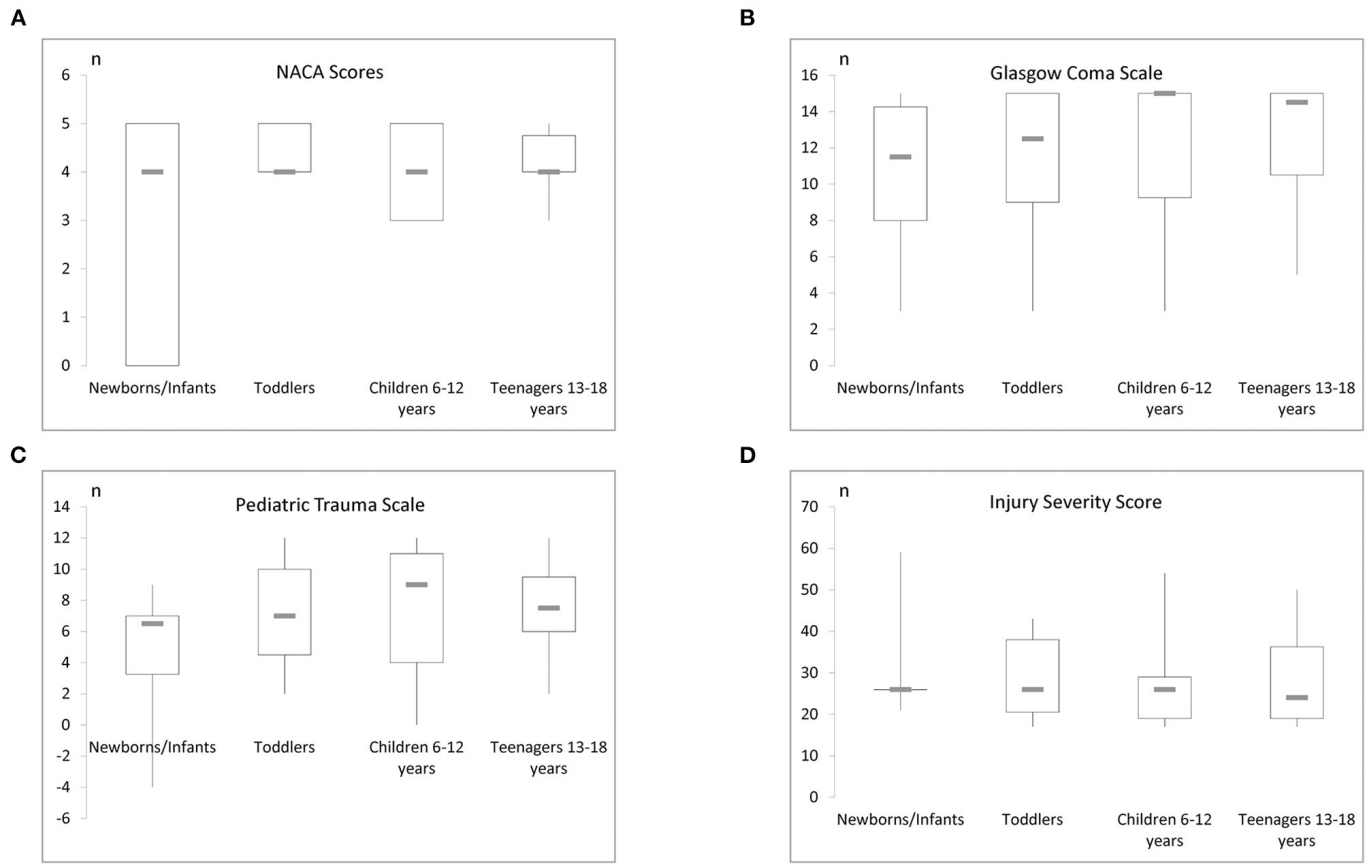
**(A)** The NACA score and its age distribution can be seen here. **(B)** This figure reveals the age distribution of the initial GCS. **(C)** This figure displays the PTS in distinct age strata. **(D)** Illustration of the age specific ISS levels in this study.

For 64 patients the NACA score was applied at the accident site during the initial assessment. NACA scores 3–5 were assessed (Table 2). There was no significant correlation between the NACA and the ISS (*p* = 0.114; *r* = 0.199). However, NACA correlated with the PTS (*p* = 0.01; *r* = 0.65). We show the age distribution of the NACA score in Figure 2A.

### Scoring With the Glasgow Coma Scale (GCS)

Assessment of the polytrauma patients at the trauma site with the GCS was performed in 90.7% of the patients and revealed a mean score of 12.4+/-3.9 (Table 2; **Figure 4**) whereas in the trauma room the GCS was assessed in 95.8% of patients and the average score was 10.4+/-5.3. Patients who were transferred from another hospital showed an average GCS of 9.1+/-5.9. No significant difference between the different sites was noted. The age distribution of the initial GCS or pediatric GCS is shown in Figure 2B. During the initial assessment, most of the patients had a GCS of 15, however, 8 patients presented with a GCS of 3. Scores between 4 and 14 were detected in the remaining children with a frequency of 1–6 for each score.

### Scoring With the Pediatric Trauma Score (PTS) and the Injury Severity Score (ISS)

Calculation of the Pediatric Trauma score found values between −4 and 12 with an average of 7 +/- 4 (Table 2; Figure 2C). According to the inclusion criteria, the ISS scores ranged from 17 to 59 with a mean score of 28+/-10 (Figure 2D).

### Evaluation of the Different Scoring Systems

ISS correlated with the PTS (*r* = −0,541; *p* < 0.01; *r*^2^ 0,293). When we compared the ISS and the GCS to the PTS by grouping the patients according to the severity of their injury, the group presenting with lower trauma scores according to the PTS (9–12) scored above a GCS of 8 and below an ISS of 50, indicating that lower PTS scores may confirm the lack of severe traumatic brain injury. The ISS and the GCS showed a great variation within the groups with lower PT scores. Patients with potentially life-threatening and life-threatening injuries according to the PTS (PTS 0-8) showed all levels of traumatic brain injuries and all graduations of ISS.

**Figure 3:**
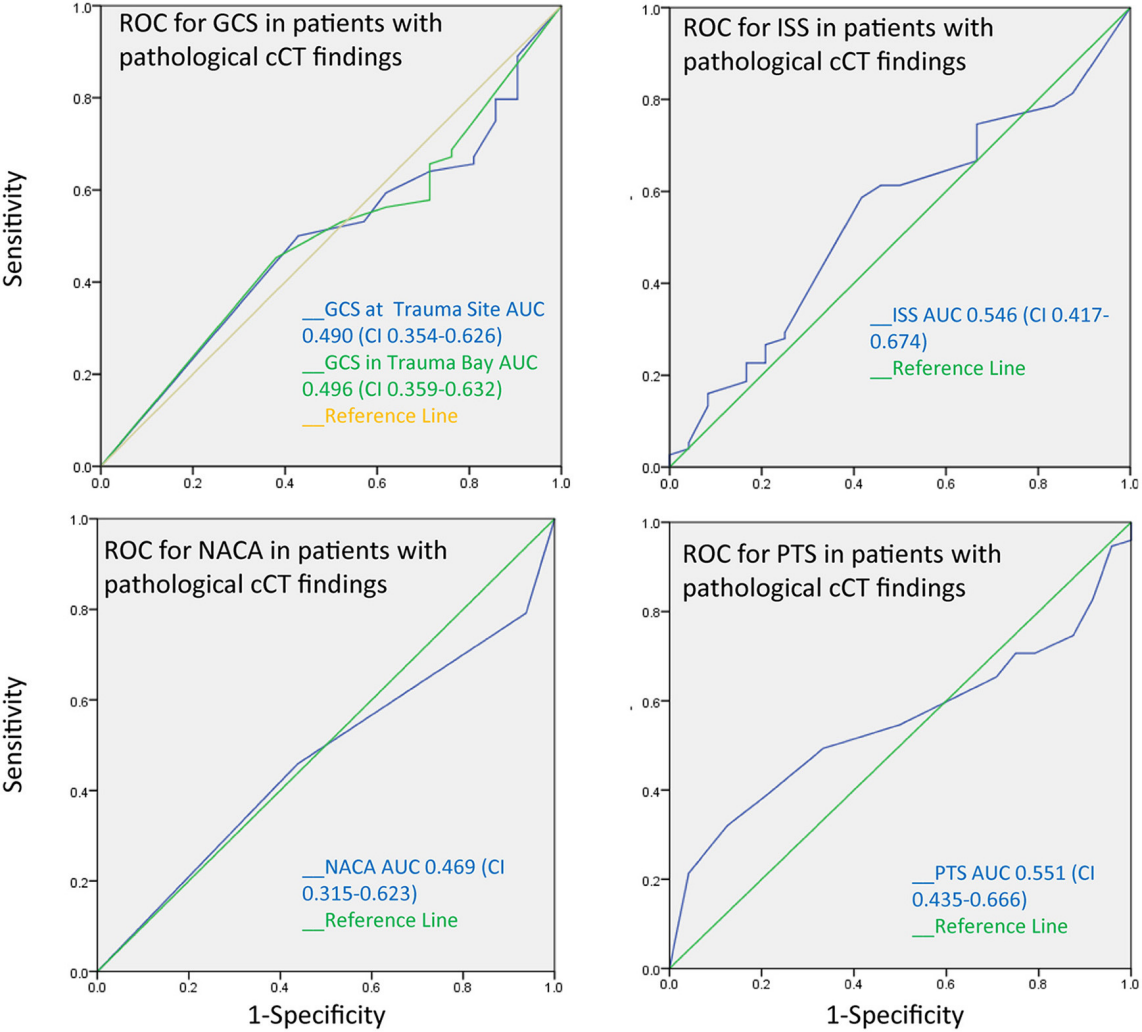
Receiver operating characteristics curves for pathological cranial CT findings. The area under the curve is near 0.5 for all trauma scores tested. Scores therefore cannot distinguish between patients with and without pathological CT findings.

**Figure 4:**
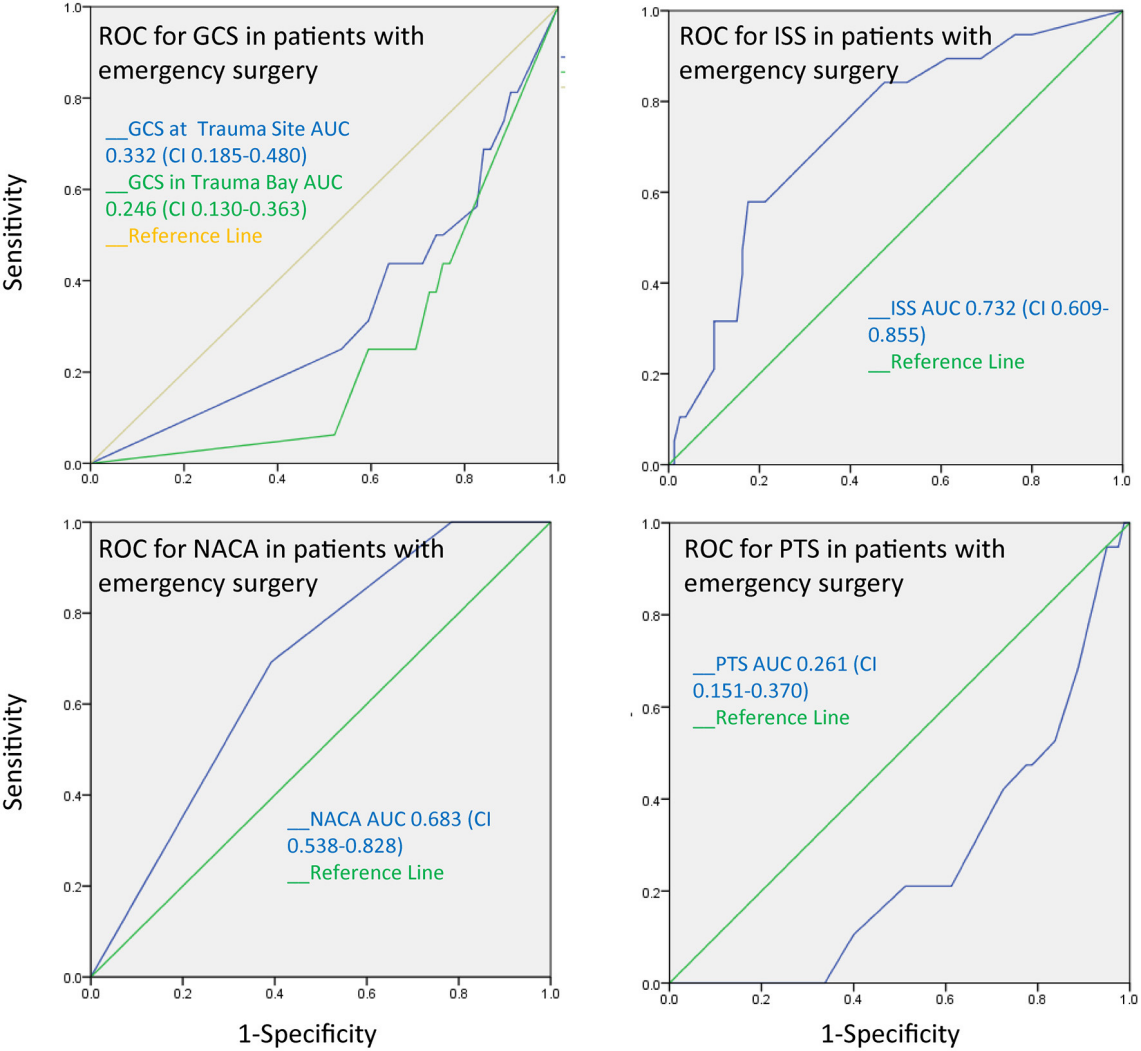
The receiver operating characteristics curve for emergency surgery after trauma demonstrates that the ISS and the NACA score can discriminate between patients who patients who underwent emergency surgery and those who did not.

**Figure 5:**
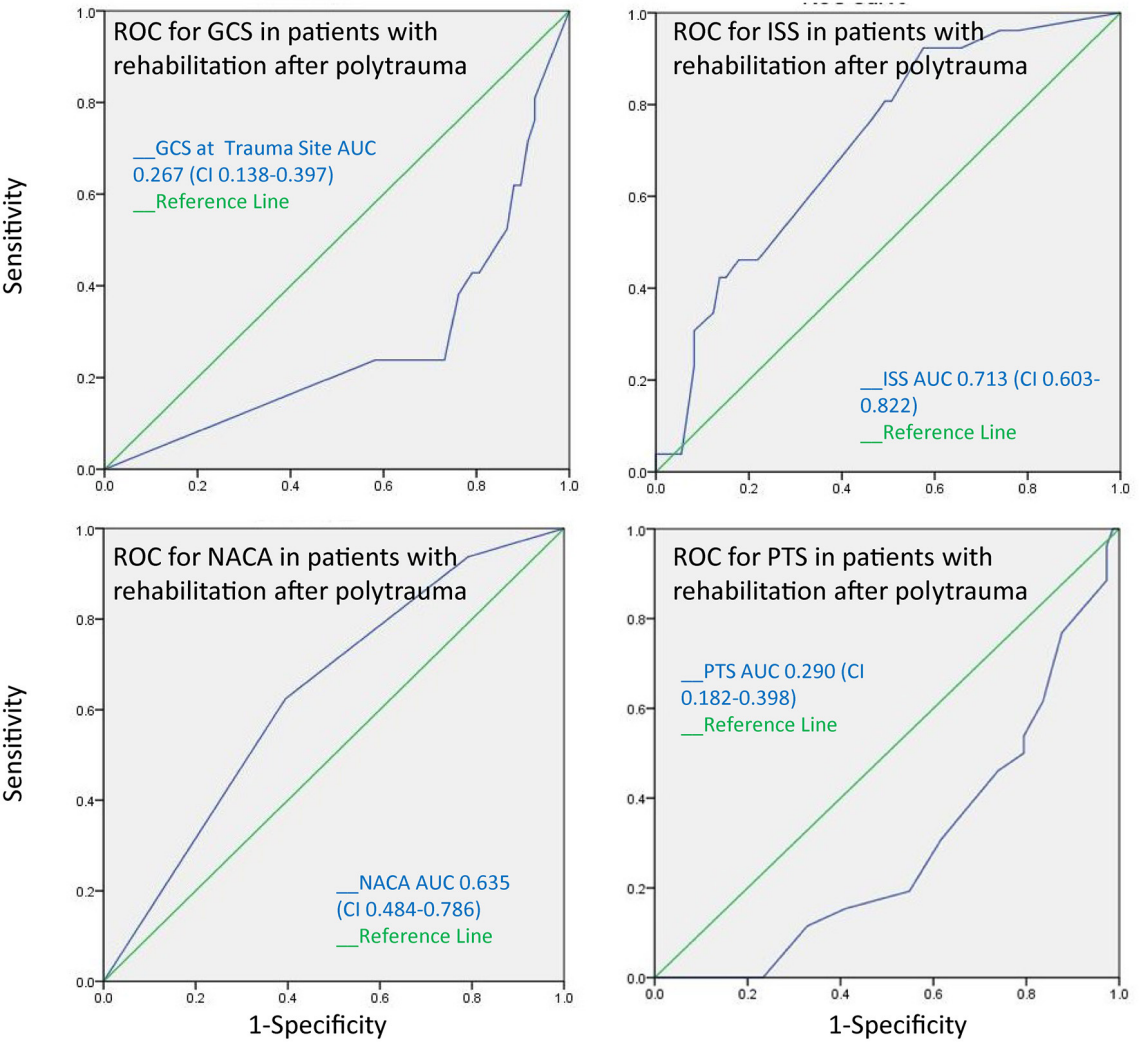
The receiver operating characteristics curve for the need of rehabilitation after polytrauma indicates that the ISS and the NACA score can discriminate between patients whose injuries were severe leading to further rehabilitation measures after acute treatment and those who were able to be discharged home without additional treatment.

The AUC for the PTS, NACA, GCS, and ISS score to discriminate between patients with and without pathological cranial computed tomography was calculated at 0.55, 0.47, 0.49, and 0.55, respectively, and therefore had no substantial predictive value (Figure 3). The AUC regarding the CT findings of the spine, thorax, and abdomen were similar, i.e., 0.58 (PTS), 0.53 (NACA), 0.55 (GCS), and 0.46 (ISS). However, the AUC for ISS (0.73), and NACA (0.68), but not for PTS (0.26) and GCS (0.332) discriminated patients who needed emergency surgery vs. those who did not (Figure 4). Notably, the AUC for ISS (0.71) and NACA (0.64), and not for GCS (0.27) or PTS (0.29) also differentiated patients who needed rehabilitation vs. those who did not which reflects the severity of the injuries and confirms our results (Figure 5).

### Treatment Duration

The median treatment duration after polytrauma was 12 days (range 0–106 days). A mean of 4 days was spent in the intensive care unit (ICU). The mean duration of intubation of the 51 intubated patients was 6 days (range 0–46 days). While the duration of intubation did not correlate with ISS (*r*^2^ = 0.00; *p* = 0.89), the duration of the intensive care did (*r*^2^ = 0.07; *p* = 0.006).

## Discussion

In this study, we analyzed different trauma scores of 97 polytraumatized pediatric patients (ISS, NACA, PTS, and GCS) and correlated them to their clinical course. The receiver operating characteristic (ROC) curves showed that ISS and NACA may better identify patients who need emergency surgery than GCS and PTS. This is surprising because the PTS was specifically designed for children. The need for rehabilitation mirrors the severity of the injury, and since the ROC curves demonstrated that ISS and NACA also better revealed patients who were sent to a rehabilitation center after polytrauma, our results were hereby confirmed.

As another interesting finding, none of the trauma scores were useful in predicting pathological CT findings in children. This implies that other clinical neurologic findings must be considered to accurately predict prognosis. Decisions surrounding neurosurgical interventions should not be based solely on trauma scores.

Additionally, we found a significant positive correlation between the ISS score and the duration of intensive care treatment in patients with an ISS over 16. However, this correlation was not significant when the group of patients with an ISS over 25 was analyzed, which may be attributable to the findings of a previous study by Brown et al. who suggest that the threshold for the ISS value should be set to 25 instead of the usual 16 for the definition of severe injury in children in order to consider the differences between adults and children (23). Therefore, in this study, the ISS score could only differentiate between different grades of severity below the value of 25. This result is in agreement with a previous study, although only adult patients were included (24). Future studies with a larger study population are needed to further elucidate this topic.

Nevertheless, we included the patients for this study according to the commonly used cutoff of an ISS > 16 and we found that the ISS predicted serious injuries leading to emergency surgery in this cohort.

The definition of polytrauma remains under discussion (25, 26). Although trauma scores are important tools for the estimation of injuries and quality management, our study demonstrates their specific limitations. The ISS has been used since 1974 and is the most extensively used trauma score to date (21, 27). The application of the ISS for the evaluation of the injured pediatric patient has been under discussion due to the physiologic and anatomical differences between adults and children (23). However, even with pediatric scores available, the ISS continues to be applied for the pediatric trauma patient (28). In our study, ISS performed better than PTS in predicting outcome or need for intervention. Therefore, in search of an optimized evaluation of the polytraumatized child, we suggest a combination of some of the currently established trauma scores. The ISS could be combined with a specifically for pediatric patients modified NACA score, which may take into account the body surface and any occurring airway problems, and with the AVPU score in order to develop a more effective pediatric score for the polytraumatized child.

The next unexpected finding in this study demonstrated that the GCS did not predict the occurrence of intracranial pathologies in cranial CTs, nor the necessity of emergency surgery. This is striking since previous studies have claimed the GCS to be predictive for traumatic brain injury (29). Although the prognostic value of GCS for adults and children has been discussed previously (30) and the sensitivity and predictive usefulness has been challenged before (31), the GCS holds its prominent role as a predictor for the outcome of traumatic brain injury (TBI) and polytrauma patients (32). Nevertheless, the severity of TBI was significantly related to the lethality (*p* = 0.0001) although this was not represented by the GCS. Possibly, the GCS may simply be too complicated in its application to produce valid, reproducible results. Some have therefore recommended the use of other consciousness scores like the “alert, verbal, pain, and unresponsive” (AVPU) scale (19). Another predictor of lethality was the seriousness of the thoracic trauma (*p* = 0.04). This is in concordance with previous articles which indicate that the lethality of the injured child increases with the occurrence of thoracic trauma (33). However, with a relatively low number of lethally injured patients in our study, further studies including a larger number of patients are needed.

### Limitations

There are some limitations in our study that should be addressed in the future. The design of the study was retrospective. The scores under investigation have different approaches since the GCS score is a physiological score whereas the ISS is an anatomical score, the PTS considers injuries, as well as body weight in order to address the characteristics of children of different ages and the NACA, regards the necessary treatment that will be required due to the injuries. Although the number of patients was relatively large for a single center study, a larger study population with more balanced patient characteristics will be needed to confirm our results. Further, a prospective design, possibly including a modified pediatric ISS score, may be derived from these results for a future study.

### Summary

Taken together, the value of trauma scores that are being used for adult patients need further prospective examination regarding their applicability in pediatric patients. In our study cohort, the trauma scores investigated were not sufficient to predict any pathological computed tomography findings following polytrauma. However, the ISS and the NACA showed acceptable values regarding the AUC of the receiver operating characteristic curves (AUC = 0.7) for the prediction of needed emergency surgery. The length of the hospital stays at the intensive care unit as another parameter of severe trauma only correlated with the ISS for patients with a score below 25. Decisions surrounding neurosurgical interventions should not be based solely on trauma scores. Overall, ISS was the best predictor of outcome in our study, which indicates that at this time, it should be used as the primary scoring system, even in pediatric patients.

## Data Availability Statement

The raw data supporting the conclusions of this article will be made available by the authors, without undue reservation.

## Ethics Statement

The studies involving human participants were reviewed and approved by Ethikkomssion der medizinischen Fakultät, LMU, Munich, Germany. Written informed consent from the participants' legal guardian/next of kin was not required to participate in this study in accordance with the national legislation and the institutional requirements.

## Author Contributions

ML designed the study and revised the manuscript. AH collected and analyzed the data. DW-K analyzed the data and wrote the paper. OM co-wrote and revised the manuscript. All authors contributed to the article and approved the submitted version.

## Conflict of Interest

The authors declare that the research was conducted in the absence of any commercial or financial relationships that could be construed as a potential conflict of interest.

## Publisher's Note

All claims expressed in this article are solely those of the authors and do not necessarily represent those of their affiliated organizations, or those of the publisher, the editors and the reviewers. Any product that may be evaluated in this article, or claim that may be made by its manufacturer, is not guaranteed or endorsed by the publisher.

## Abbreviations

ISS: Injury Severity Score
PTS: Pediatric Trauma Score
NACA: National Advisory Committee for Aeronautics
GCS: Glasgow Coma Score
ROC: receiver operator characteristic
AUC: area under the ROC curve
ANOVA: Analysis of Variance
CT: computed tomography
FAST: Focused abdominal sonography for trauma
TBI: traumatic brain injury
CPR: cardiopulmonary resuscitation
ICU: intensive care unit
AVPU, alert, verbal, pain: unresponsive.
